# LLPS *vs.* LLCPS: analogies and differences

**DOI:** 10.1039/d2sm01455f

**Published:** 2023-02-14

**Authors:** Paride Azzari, Raffaele Mezzenga

**Affiliations:** a Department of Health Sciences and Technology, ETH Zürich Schmelzbergstrasse 9 8092 Zürich Switzerland raffaele.mezzenga@hest.ethz.ch; b Department of Materials, ETH Zürich Wolfgang Pauli Strasse 10 8093 Zurich Switzerland

## Abstract

We compare the process of Liquid–Liquid Phase Separation (LLPS) of flexible macromolecular solutions, with the Liquid–Liquid Crystalline Phase Separation (LLCPS) of semiflexible polymers and rigid filamentous colloids, which involves the formation of a liquid phase that possesses a directional alignment. Although the observed phase separation follows a similar dynamic path, namely nucleation and growth or spinodal decomposition separating two phases of dilute and concentrated compositions, the underlying physics that defines the theoretical framework of LLCPS is completely different from the one of LLPS. We review the main theories that describe the phase separation processes and relying on thermodynamics and dynamical arguments, we highlight the differences and analogies between these two phase separation phenomena, attempting to clarify the inner mechanisms that regulate those two processes. A particular focus is given to metastable phases, as these intermediate states represent a key element in understanding how phase separation works.

## Abstract 2

We compare the process of Liquid–Liquid Phase Separation (LLPS) of flexible macromolecules, with the Liquid–Liquid Crystalline Phase Separation (LLCPS) of rigid fibrils, which involves the formation of a liquid phase with a directional alignment.

## Graphical abstract

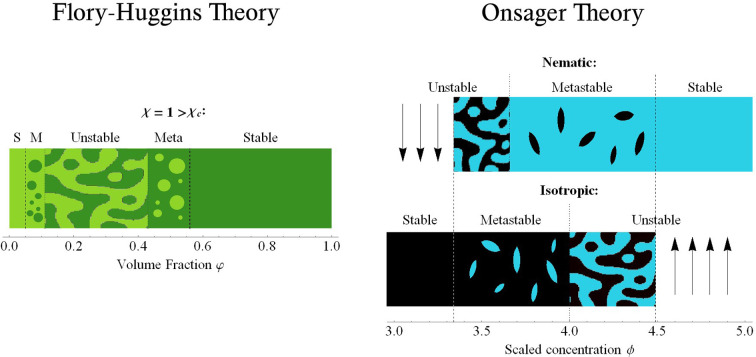

## 1 Introduction

The process of phase separation is ubiquitous in nature. A multitude of physical phenomena involve this thermodynamical process, where an initially homogeneous multicomponent mixture evolves into two or more phases with different physical properties.^1,2^ Phase separations in liquids have grown a strong interest in recent years, since many branches of science, such as biotechnology, food and health sciences deal with liquids and their phase transitions.^3–5^ The process of liquid–liquid phase separation (LLPS) is particularly evident in systems composed of solvent and solute, where a homogenous phase separates into two distinct phases: one more diluted and one more concentrated.^6,7^ This event is strongly influenced by the type of interactions between the components and external variables, such as temperature or pressure.^7–10^ This process of LLPS has been shown to be an important aspect in biological processes, due to its involvement in a wide range of phenomena in the cellular and extracellular environments.^11,12^ Many interesting phenomena are governed by liquid–liquid phase separation, *via* the upconcentration of a certain key molecule that triggers the formation of assembled structures or granules, also called condensates.^11^ Many intracellular components are organized into organelles, which can be membranebound or membraneless.^13^ The membraneless organelles behave and coalesce like liquid droplets and are formed by LLPS.^14^ Liquid–liquid phase separation phenomena have served life to develop a structured cellular infrastructure and to deliver biochemical functions.^15^ Through the formation of concentrated domains of biomolecules, many biological processes are enabled or enhanced: from transcriptional activation and regulation of RNA,^16^ to its remodeling and stabilization,^17^ the formation and regulation of granules and condensates,^15^ the process of stress signaling^18^ and even SARS-Covid-2 viral assembly^19^ are all different examples of how this type of phase separation induces a wide range of biological effects. The process of liquid–liquid phase separation is usually rationalized through the Flory–Huggins theory,^20^ which describes the phase separation of a mixture of macromolecules as a thermodynamic interplay between the entropy contributions of mixing and the interaction energy between the different species of molecules.^21^

In the Flory Huggins treatment of LLPS, the entropy favors mixing as it introduces degrees of freedom *via* the position (center of mass) and configuration of macromolecules and it is intrinsically related to the capability of these components to adopt different configurations in a solution. However, when the macromolecules become progressively more rigid, these degrees of freedom are lost down to the level that, for infinite rigidity, their configuration in solution is described only by their positional and angular distribution.^21^ In this limit, the Flory–Huggins theory no longer holds and the traits of LLPS change fundamentally into a distinct phase separation mechanism called liquid–liquid crystalline phase separation (LLCPS). The main difference between LLCPS and LLPS is that the most concentrated phase among the two phase-separated is now characterized by an orientational order of the rigid macromolecules, that is, is controlled by the presence of liquid crystalline interactions. Ultimately, it is the ratio between the contour length of the polymer *L* and its persistence length *L*_P_ that determines whether phase separation occurs *via* LLPS (*L* ≫ *L*_P_) or LLCPS (*L* < *L*_P_).^22,23^ Liquid crystals are an intermediate state of matter, which show liquid mechanical properties, and an ordered structure typical of crystalline systems, where the order is exhibited in the alignment of the liquid crystal molecules. This order makes possible the formation of nematic phases, that is anisotropic phases in which all the liquid crystalline molecules are aligned along a specific direction.^24^ These molecules, which form anisotropic phases, are called nematogens, and they are usually elongated rigid molecules. Liquid crystalline solutions, at certain concentrations, can trigger a process of phase separation.^25^ From a completely isotropic system, the solution will form domains of aligned fibrils and eventually phase separate into a nematic phase.^26^ These nematic domains are named tactoids. Liquid crystalline tactoids have been intensively studied: the structure and nematic field have been analyzed and interpreted in detail;^26,27^ however, only recently the formation of tactoids through the process of nucleation and growth, has been formalized for liquid crystals.^22^ In the phase of growth, nematic tactoids increase in size to reach thermodynamic equilibrium and attain a macroscopically phase-separated system.^28^ Liquid crystalline phases have been strongly used in health science and biomedical applications^29^ and in pharmaceutical technologies.^30^ The most typical biological macromolecules, that self-assemble in liquid crystalline structures, are collagen, actin, cellulose and filamentous viruses.^31^ In recent years, nematic and cholesteric phases have been reported in amyloid fibrils.^32^ These fibrils, formed from β-sheet aggregates, have revealed unique self-assembly capabilities, displaying an unprecedented palette of morphologies in the liquid crystalline phases.^26^ Amyloid fibrils have also grown a strong interest in many disciplines: from health sciences to food science, for their importance and their role in biological processes.^33,34^ Moreover, amyloid fibrils have reached a wider audience, since their functional characteristics, made them a key element in various fields from material science to bionanotechnology.^32,34,35^ Before these positive roles, amyloid fibrils were, however, initially known for their association with neurodegenerative pathological conditions, for example, Parkinson's disease and Alzheimer's disease.^36^ Therefore, understanding the processes behind the emergence of liquid crystalline phases, and revealing the mechanisms behind LLCPS in comparison to standard LLPS, may even give a stronger foundation to understand the nature and formation of amyloid deposits, and eventually devise successful treatments for these conditions.

To date, the processes of liquid–liquid phase separation and liquid–liquid crystalline phase separation are not well differentiated. The purpose of this work is to analyze the mechanisms that are underlying the two different processes and understand their similarities and differences.

## 2 Phase separation generalities

We want to start by recalling the general traits of phase separation from a thermodynamical point of view, and to review the determining of the unstable phases and equilibrium phases of a system composed of solvent and solute. Independently from the molecular and physical traits of the solute. In a solution of volume *V* and given volume fraction *φ*, we can define a free energy *F* of the form

1 *F* = *Vf*(*φ*),

where we assume for simplicity that *k*_B_*T* = 1. We can write the thermodynamic potentials as2
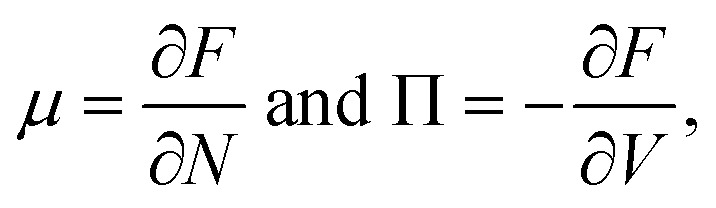
where *μ* is the chemical potential and Π is the osmotic pressure. In the solution, we consider a small subvolume Δ*V*. If in this small subvolume the solute concentration goes up, from *φ* to *φ* + *ε*, due to a random fluctuation in the solute, this event will induce a change in the osmotic pressure:

3 Π_Δ*V*_(*φ* + *ε*) − Π_*V*_(*φ*) ∼ *εφf*′′(*φ*)

This expression is obtained, for very small *ε*, from Π = *f*′(*φ*)*φ* − *f*(*φ*). Therefore, we can deduce that the concavity of *f* influences the volume flow, generated by this pressure imbalance. When *f*′′ is greater than zero, any fluctuation in the system is balanced by an increase in pressure, which forces a volume flow that restores the initial condition, therefore this is called a stable state. Otherwise, when *f*′′ is negative, the system is unstable. The given subvolume has a pressure imbalance that amplifies the volume difference. This thermodynamical instability is the basis of phase separations.^37^

For systems where *F* is of the form of eqn (1), we can define the spinodal line as the points where *f* changes concavity, *i.e. f*′′(*φ*) = 0. The spinodal line delimitates the spinodal region where *f*′′ is negative, in which the system is in an unstable state. From this condition, the system will evolve into stable states through a phase separation process called spinodal decomposition.^38^

If the fluid phase separates into two different phases, we want to understand the equilibrium conditions for the coexistence of these two stable states. Let *F*_tot_ be the sum of two subsystems in two different phases:

4 *F*_tot_ = *V*_1_*f*(*φ*_1_) + *V*_2_*f*(*φ*_2_)

where *V*_1_ + *V*_2_ = *V* and *V*_1_*φ*_1_ + *V*_2_*φ*_2_ = *Vϕ*. By minimizing the above equations with the given constraints, we obtain that the total free energy is minimized only when the thermodynamic potentials in the two phases are equal

5 *μ*_1_ = *μ*_2_ and *Π*_1_ = *Π*_2_.

Phase coexistence can be identified as the states sharing the same potentials. When the chemical potential and pressure are not balanced, the system will spontaneously evolve to restore thermodynamic equilibrium. The states where the potentials are equal form the so-called coexistence or binodal lines. The system can exist in two different phases, if the binodal conditions are met.^21^ For a more indepth analysis, see ref. 39.

## 3 The Flory–Huggins theory

For a two-component system of solute and solvent, the free energy density is usually described with the form given by the Flory–Huggins model:^40^6
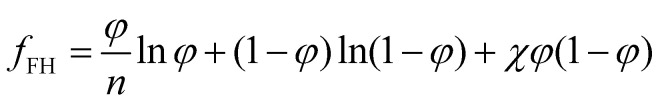


**Fig. 1:**
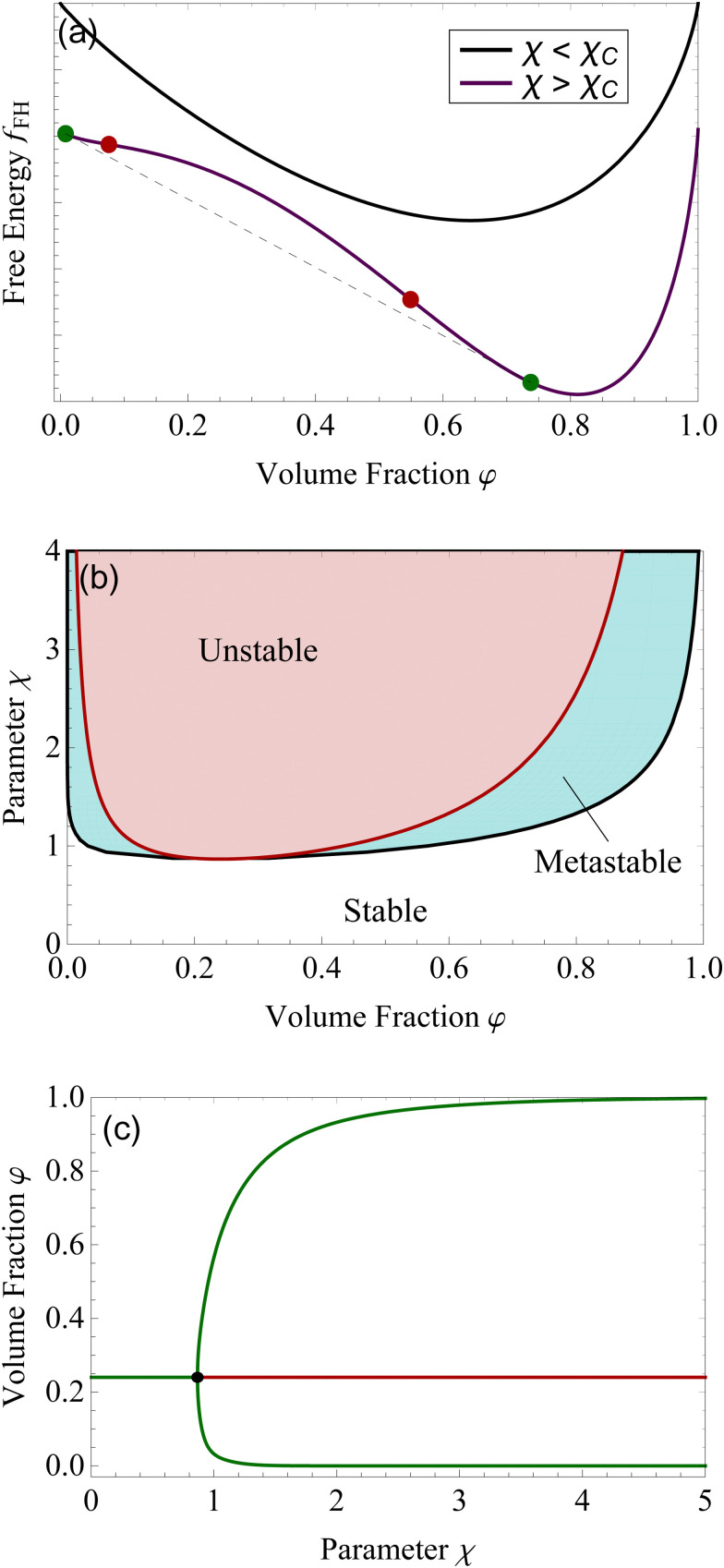
For the Flory–Huggins model, panel (a) represents two behaviors of *f*_FH_. When *χ* < *χ*_c_ (black line) and *χ* > *χ*_c_. The red dots are the spinodal points, the green ones the binodal. We plotted in panel (b) the binodal line (black) and the spinodal line (dark red). The area enclosed by the spinodal line is the unstable region (red area), the stable region is in white, and the metastable is represented by the cyan area, between the binodal and spinodal curves. Panel (c) shows the bifurcation plot, with the critical point at *χ*_c_ = 0.87 for *n* = 10. The green lines are stable points, the red one is the unstable branch.

Although for macromolecules, *n* is of the order of 10^3^ or 10^4^,^21^ here we consider *n* = 10. This parameter influences the symmetry of the free energy, but not the topology, in which we are interested. In contrast, the parameter *χ*, strongly influences the shape of the curve *f*_FH_. For each value of *χ*, we can determine the equilibrium points and the stable and unstable zone of the system, as described before. By varying *χ* we can identify two different behaviors of the free energy, as shown in Fig. 1(a). Below a critical *χ*_c_, *f*_FH_ is always convex, while above that value a concave region is formed, *i.e.* in between the red points in Fig. 1. This concave region is the spinodal region. The green points in Fig. 1(a) are those giving equality of the chemical potential and osmotic pressure, see eqn (5), that is the binodal points. The critical value of the parameter *χ* can be obtained by minimizing the spinodal, *i.e.*
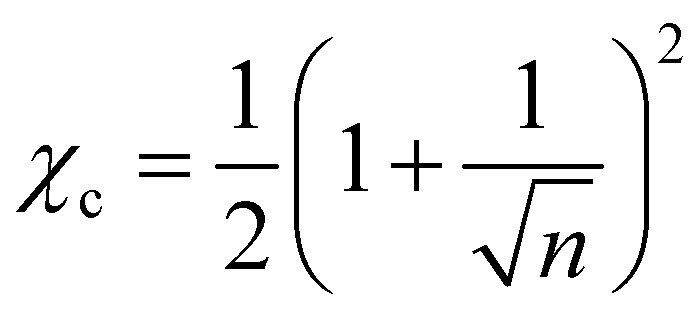
, in the case of *n* = 10, we have *χ*_c_ = 0.87. This parameter is a factor determined by the interaction energies between the components and the temperature. Similarly, the critical concentration can be obtained as 
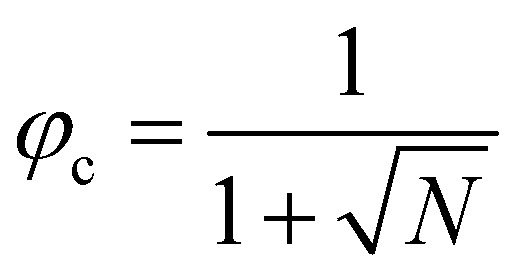
, for *n* = 10, *φ*_c_ = 0.24. For a more in-depth analysis see the references.^41–43^

The spinodal and binodal lines for varying *χ*, have been plotted in Fig. 1(b). The stable region is in white. Each point in the white area is stable and will not evolve or phase separate. The red zone is the unstable region, where 
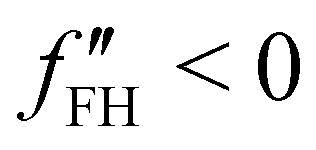
, with the spinodal curve surrounding it (highlighted in dark red). The spinodal line generates a miscibility gap, solutions with certain volume fractions are not stable, and for high values of *χ*, only low or high concentrations are stable and do not phase separate. The binodal line (black) is encircling the spinodal region. Solutions with volume fractions on this line at fixed *χ*, can coexist in the same system. The region between the binodal and spinodal line is a metastable zone, here phase separation is possible, but there is an energetic barrier to overcome.^44^ Any metastable solution will eventually nucleate and grow droplets to reach a stable phase. The spinodal and binodal lines meet in the critical point at *χ*_c_ = 0.87 for *n* = 10.

We want to study the same system from a dynamical system point of view. Following a solution of initial concentration *φ* = 0.24, for *χ* lower than *χ*_c_. By slowly increasing the parameter *χ*, the solution will remain in a stable state until the critical point is reached *χ*_c_, see Fig. 1(c). For higher values, the volume fraction of 0.24 becomes unstable, and the solution will divide into two different phases, a more concentrated one (upper green branch) and a more diluted one (lower green branch). The plot shown in Fig. 1(c) is called bifurcation diagram, where the bifurcation point is the critical point for the Flory–Huggins model, *χ*_c_ = 0.87 and *φ* = 0.24. This type of bifurcation is called supercritical pitchfork bifurcation,^45^ a type of bifurcation that can be observed in other physical systems, such as ferromagnets, and other second-order phase transitions.^39,46^

## 4 The Onsager theory

The Flory–Huggins model applies ideally to flexible macromolecules, for which the conformational entropy results in a simple sum of logarithms as in eqn (6). Now, we focus on hard rigid rods: polymeric fibrils whose persistence length *L*_P_ is much greater than their contour length *L*.^47^ In a solution of hard rods of volume fraction *φ*, we can define a scaled concentration 
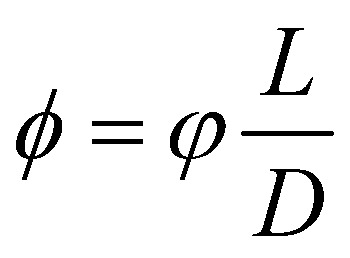
, where *D* is the diameter of the fibril. According to Onsager's theory,^48^ the total free energy *F*_OT_ of a solution of hard rods has the form7
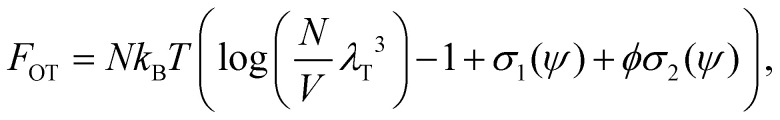
where *λ*_T_ is the thermal wavelength. However, in the analysis, we will refer to the excess free energy density *f*_O_ as the quantity8
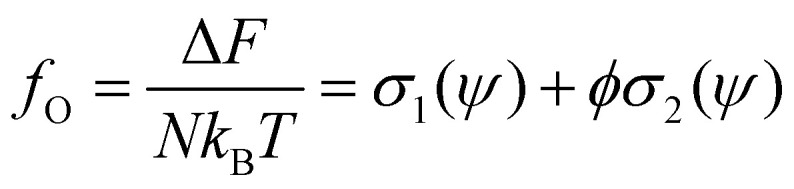
which represents the difference in free energy between a solution of hard rods and a perfect gas, per unit of solute. The functions *σ*_1_††
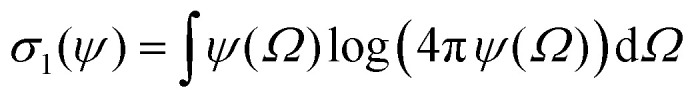
 and *σ*_2_‡‡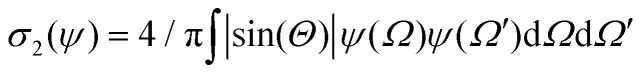 depend of the orientation distribution of the rods *ψ*. When *ψ* = 1/4π the fluid is said to be isotropic, where any orientation is equiprobable.^48^ From the orientation distribution *ψ*, it is possible to calculate a degree of alignment along a given direction, this measurement is called the order parameter9
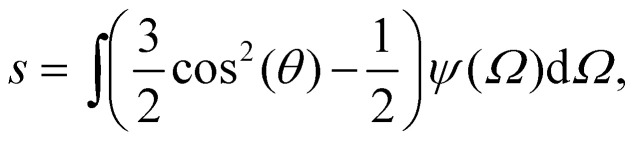
when *ψ* = 1/4π, *s* = 0, therefore there is no alignment, while for *s* > 0 the rods are aligned and therefore, they form a nematic state. For *s* = 1 all the rods are parallel to each other.

Using the Onsager trial function, we can evaluate *σ*_1_ and *σ*_2_, and compute the order parameter *s* that minimizes the free energy *f*_O_ at each concentration *ϕ*. From the phase separation generalities discussed before, we can calculate the thermodynamic potentials (chemical potential and osmotic pressure) for eqn (8). Equating them for the isotropic and nematic case we obtain the two values of *ϕ*_I_ = 3.34 and *ϕ*_N_ = 4.49. The functions *σ*_1_ and *σ*_2_ are integral functions of the orientation distribution *ψ*, which in turn depends on the order parameter *s*. Minimizing eqn (8) with respect to *s*, to obtain the equilibrium order parameter for different values of *ϕ* cannot be done analytically. Only numerical solutions are possible, as discussed in detail in ref. 48. However, to better understand how these quantities interact to form nematic and isotropic phases, a simplified approach is given in the appendix. A more detailed discussion on the Onsager functional can be found in ref. 24. For values of *ϕ* below *ϕ*_I_, the isotropic state is the only possible stable state, while for values of *ϕ* greater than *ϕ*_N_, the only available state is the nematic one. The values of *ϕ* in between those two values form the coexistence window, where both nematic and isotropic phases are coexisting together. Phase separation is only possible between these two values.

**Fig. 2:**
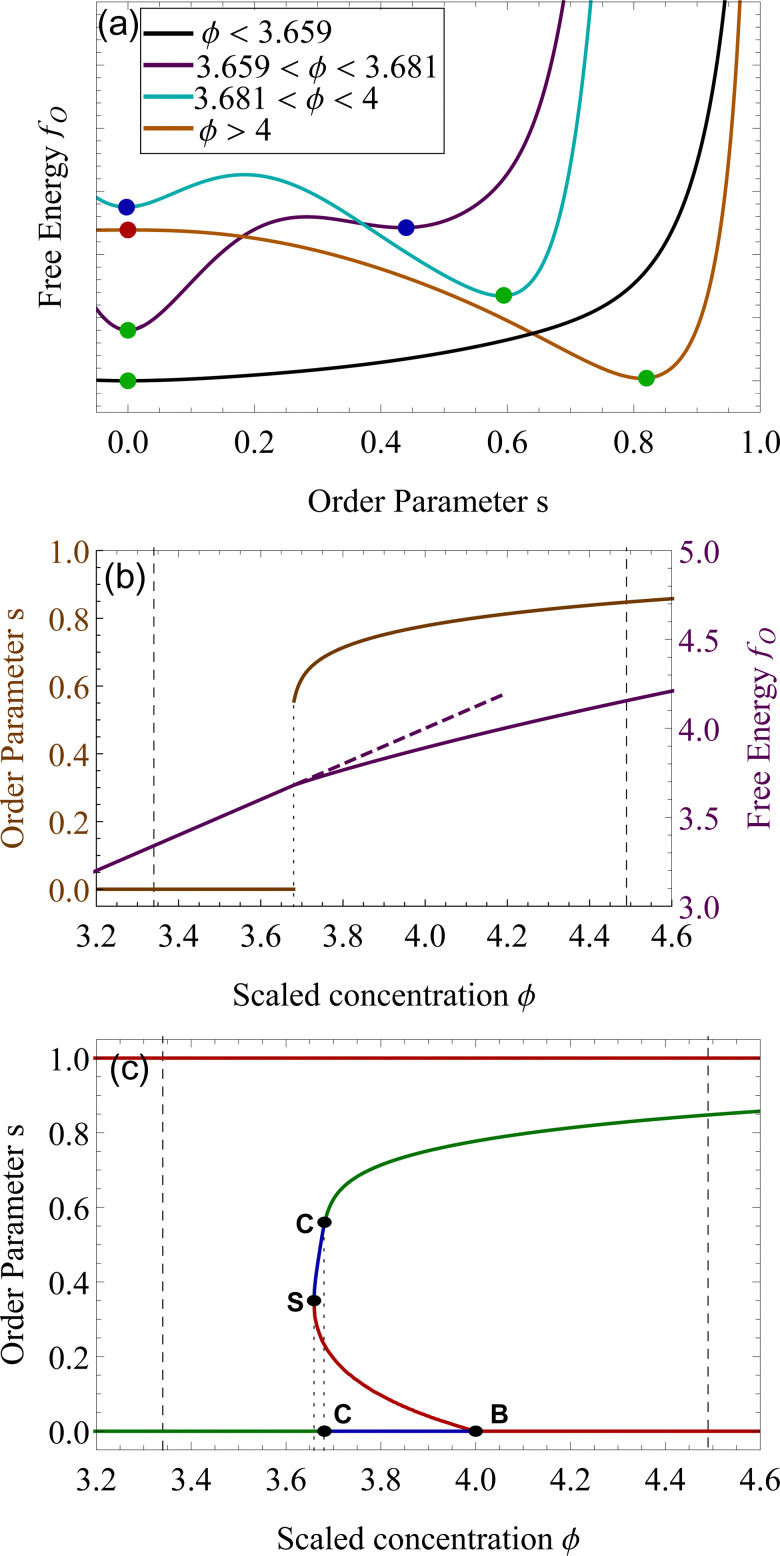
Panel (a). Excess free energy density *f*_O_ as a function of the order parameter *s* for different concentrations *ϕ*. The green dots represent stable equilibria, absolute minima of *f*_O_, the blue dots metastable equilibria, local minima of *f*_O_, while the red dot is an unstable state, a local maximum of *f*_O_. We plotted in panel (b) the order parameter *s* (orange) and the free energy (purple), as a function of the scaled concentration *ϕ*. The first-order transition from isotropic to nematic phase is particularly evident at *ϕ* = 3.681. Panel (c) shows the stable (green), metastable (blue) and unstable branches (red) of the Onsager theory. The dashed lines are the phase equilibria at *ϕ*_I_ = 3.34 and *ϕ*_N_ = 4.49. The highlighted points S, C, and B, refer to the bifurcations points of the Onsager Theory, as described in the text. The point S is the saddle-node bifurcation at *ϕ*_S_ = 3.659, *C* represents the critical point at *ϕ*_C_ = 3.681 and B the trans-critical bifurcation at *ϕ*_B_ = 4.

We want to investigate the thermodynamic stability of the two phases in the coexistence window. We start by analyzing the free energy landscape as a function of the order parameter *s*, for varying concentration *ϕ*. We identified four different scenarios that are summarized in Fig. 2(a). For *ϕ* lower than 3.659, the state *s* = 0 is the only minimum of the system, Fig. 2 (a, black). At *ϕ*_S_ = 3.659 a minimum appears in a nematic state where *s* > 0. This state remains metastable, *i.e.* a local minimum, until *ϕ*_C_ = 3.681. For concentrations higher than this number, this nematic state becomes the absolute minimum, that is the equilibrium state of the system, while the isotropic, changes into a (meta)stable one. This critical concentration *ϕ*_C_ is a first-order phase transition in the alignment *s*. This is also shown in Fig. 2(b), where the discontinuity in the slope of *f*_O_, clearly underlines this phase transition from isotropic to nematic, by an increase in the concentration.^24^ At a concentration higher than *ϕ* > 4, the isotropic state changes from a minimum of *f*_O_ to a maximum, therefore, the isotropic state becomes unstable from *ϕ* > 4. This is a bifurcation point for the isotropic state *s* = 0.^49^ The position of these critical points of *f*_O_ is depicted as lines in Fig. 2(c), for changing *ϕ*. The horizontal axis where *s* = 0 shows the three different stability attained by the isotropic state. We now focus on the nematic state, where *s* > 0. This state has an absolute minimum only from *ϕ* > *ϕ*_C_ = 3.681. For values lower than *ϕ*_C_, up to *ϕ*_S_ = 3.659 the nematic state is not the absolute minimum, but still a local minimum of *f*_O_. The free energy *f*_O_ diverges when *s* = 1, therefore the perfect parallel alignment is unachievable, according to this formulation. For values of *ϕ* lower than *ϕ*_S_, there are no stationary points of the free energy *f*_O_. This concentration is the first bifurcation of the Onsager model, called saddle-node bifurcation.^45^ For *ϕ* lower than *ϕ*_S_, no nematic states are present neither stable nor unstable. At *ϕ*_S_ two states, a (meta)stable one and an unstable one are created. The unstable branch eventually connects to *ϕ*_B_ = 4, forming another bifurcation, called trans-critical bifurcation.^45^ As in Flory–Huggins, Onsager's theory predicts two coexisting phases for 3.34 < *ϕ* < 4.49. That this is possible can be appreciated by looking at the curves of Fig. 2(a), for example at concentrations 3.659 < *ϕ* < 4, where the two minima observed for the free energy for two differently ordered states (*s* = 0 isotropic and *s* > 0 for the nematic) bear full analogy with the two minima of the Flory–Huggins free energy leading to the equilibrium between a diluted and concentrated phase (see Fig. 2(a)). Contrary to the Flory–Huggins theory, however, Onsager's theory shows a richer palette of bifurcations.^50^ The saddle-node bifurcation is typical of first-order phase transitions, while the trans-critical bifurcation can be seen in laser threshold systems.^51^ The joint diagram of Fig. 2(c), which includes the two bifurcations, represents a typical pattern that can be observed in other physical systems that undergo hysteresis processes, like magnetic hysteresis.^52^ Starting from an initial stable isotropic state, by increasing the concentration, when we reach *ϕ* = 4 the system will spontaneously evolve towards the nematic branch. Similarly, bringing back the concentration at *ϕ* lower than 4, we would still be on the stable nematic branch, at the concentration lower than 4. However, such an effect would be extremely difficult to observe experimentally, since concentrations in these values would eventually undergo nucleation and growth.^22,25^

## 5 Analogies and differences

The two processes of liquid–liquid phase separations induce similar phase separations dynamics such as spinodal decomposition and nucleation and growth. However, as shown above, the underlying physics that drives both phenomena is completely different. The Flory Huggins theory relies on the interplay between interaction energy between the different components and the entropy of the mixture. The free energy from eqn (1), can be rewritten as

10 *f*_FH_ = Δ*S*(*φ*) + *χ*Δ*U*(*φ*).

**Fig. 3:**
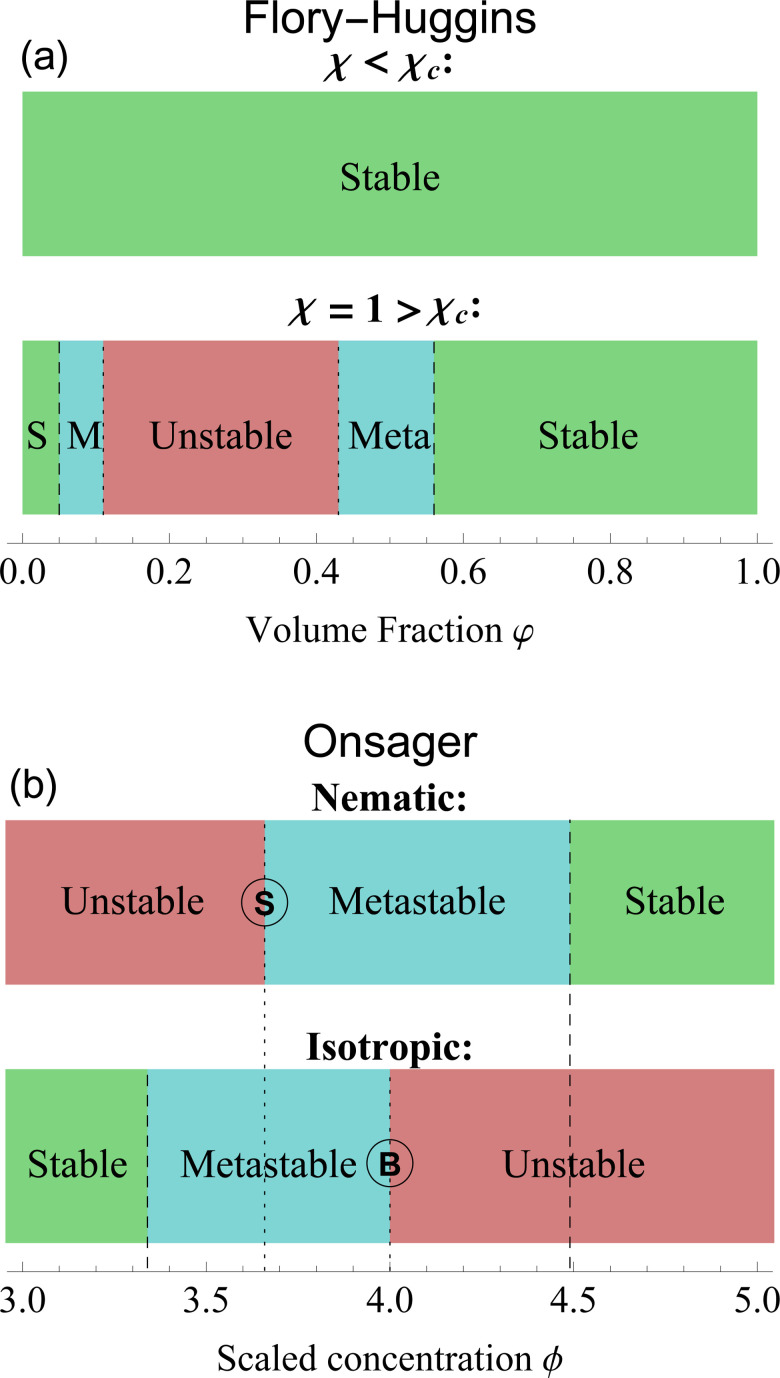
Stability diagrams for the Flory–Huggins theory (a) and the Onsager theory (b). The former shows an unstable state, surrounded by metastable (M) and stable (S) states only for *χ* > *χ*_c_ = 0.87. For this plot, we set *n* = 10 and *χ* = 1. The latter has a stability divided by isotropic and nematic phases. Nematic phases are stable for *ϕ* > 4.49 and unstable for *ϕ* < *ϕ*_S_ = 3.659. Isotropic ones, on the contrary, are stable for *ϕ* < 3.34 and unstable for *ϕ* > *ϕ*_B_ = 4. In between those values, the states are metastable. A complete analysis of the differences is in Section 6. The letter S and B identify the respective bifurcation, as shown in Fig. 2.

With this formulation, the free energy components are much more evident. The entropy Δ*S* and internal energy Δ*U* depend on *φ*, while *χ* is a parameter that balances the two contributions. For low values of *χ* the entropy dominates the system: for every volume fraction *φ*, the solution is stable; on the other side, for values of *χ* higher than the critical value *χ*_c_, the energy contribute dominates the free energy and the interaction between the components generates a miscibility gap. Volume fraction values between the spinodal points become unstable and will eventually phase separate into two distinct phases. Only high and low volume fractions are accessible, therefore LLPS forces the system into an up-concentrated phase and into a more diluted one. With the metastable zone surrounding the unstable region. The stability diagram of the Flory–Huggins theory is shown in Fig. 3(a).

Onsager's theory relies on a pure interplay between entropic contributions.^24,48^ The free energy from eqn (8) can be regrouped as

11 *f*_O_ = Δ*S*_or_(*s*) + *ϕ*Δ*S*_ex_(*s*)

The two entropy addenda represent the orientational and excluded volume contributions. Contrary to the Flory–Huggins case, in the Onsager's model we minimize the free energy *f*_O_ with respect to the order parameter *s*, while the concentration *ϕ* is the balancing factor between the two terms. While in the previous case, by changing the interaction *χ*, it is possible to split the concentration of the system *φ* into two different concentrations corresponding to the new phases, in the Onsager model, by changing the concentration *ϕ* it is possible to split the system into two new phases with different order: isotropic and nematic. Therefore, in Onsager, the composition plays an analogue role than the *χ* in the Flory–Huggins model; reversely, the order parameter *s* in Onsager plays a similar role of the concentration in *f*_FH_, as independent parameter (comparing eqn (10) and (11)). This similarity is best grasped by tilting by 90° the Onsager diagram of Fig. 2(c), which immediately starts to show similarities with the binodal lines of the Flory–Huggins theory in Fig. 1(b).

In Fig. 3(b), we plotted the stability diagram for the Onsager theory, divided into nematic and isotropic phases. The stable states exist only left of 3.34 for the isotropic state and right of 4.49 for the nematic state. The isotropic state becomes unstable for a value greater than *ϕ*_B_ = 4. While for nematic alignments, the unstable branch ends at *ϕ*_S_ = 3.659. Nematic and isotropic alignments induce a miscibility gap in hard rods system, where only concentrations below *ϕ*_I_ = 3.34 are stable in an isotropic single phase, and concentrations higher than *ϕ*_N_ = 4.49 are stable in an aligned single phase. In between these two compositions, the system only exists in a two-phase heterogeneous state.

## 6 Metastable states

In the context of thermodynamics, metastable states are defined as intermediate equilibrium states, in which the system is not in the least energy configuration.^44^ Examples of this occurrence are the nematic and isotropic states, outside of the equilibrium zones shown in Fig. 2(b). For values of the scaled concentration between 3.659 and 3.681 the nematic state is in a metastable state, while for 3.681 < *ϕ* < 4 the isotropic is metastable. This is metastability in the order parameter *s*. From an analytic point of view, this metastability is obtained as a local equilibrium of the free energy *f*_O_ at fixed concentration *ϕ*, which is not the global minimum. These metastable states are stable under small fluctuations, but not globally stable.^53^ Looking at the Onsager free energy eqn (8), between the absolute minimum and the local minimum there is an energy barrier, that determines the stability of these metastable states. High amplitude localized fluctuations can trigger a phase transition from the metastable state to the stable configuration, going from the isotropic to the nematic state (or *vice versa*), when the concentration is in the ranges listed above.^54^ For LLCPS, this type of metastability is pictured in Fig. 2(a) and (c).

**Fig. 4:**
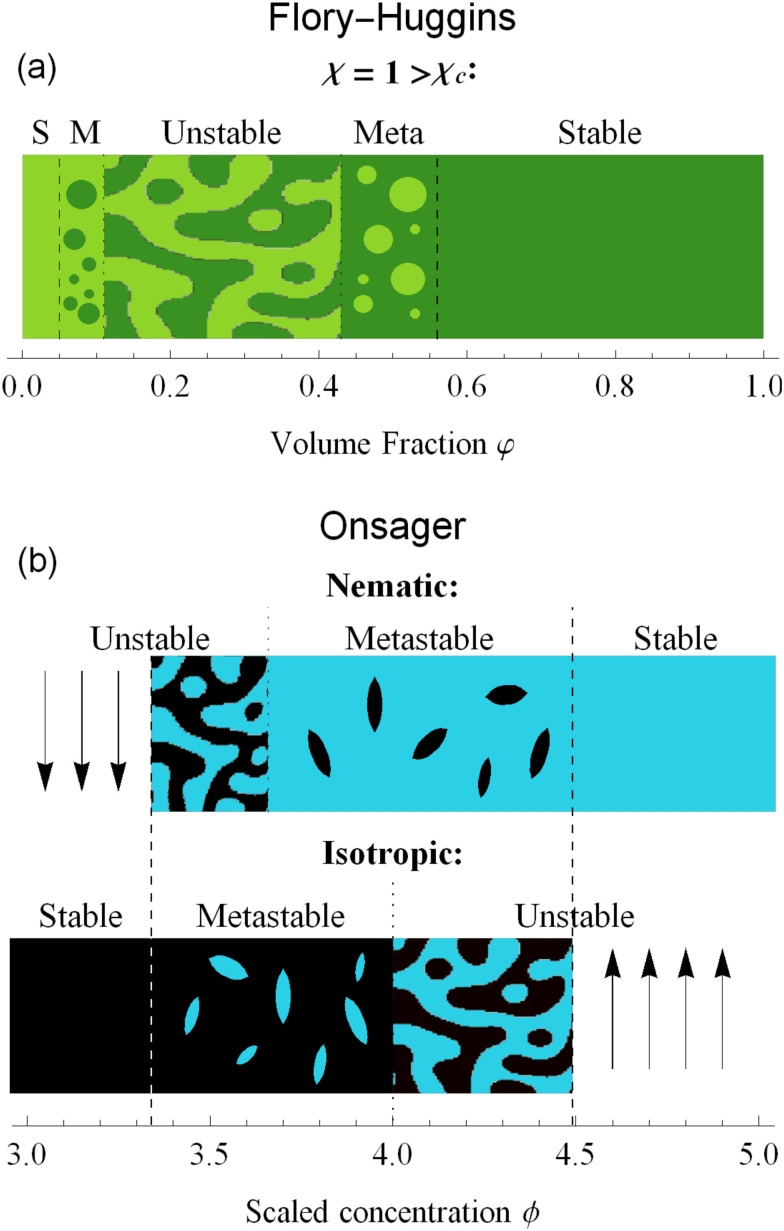
The dynamics of phase separation depends on the initial condition of the system. For Flory–Huggins, spinodal decomposition happens for values in the unstable zone, while in the metastable (M) zone nucleation and growth of droplets brings the system to equilibrium. For Onsager theory, the status of the system depends not only on the concentration, but also in the alignment. Isotropic phases will nucleate nematic tactoids in the metastable zone, while nematic phases will nucleate isotropic ones. In the unstable regions, spinodal decomposition follows.

Another type of metastability occurs in the phase coexistence window. In the Flory–Huggins model, metastable states surround the spinodal region and are delimited by the binodal lines. While binodal lines determine the coexistence, for values of the concentrations that range in the metastable zone, the difference in potentials is such that the concentrated phase has a lower potential than the diluted phase, creating an ‘up-hill’ diffusion, where the solute diffuses from the low-concentration to the high-concentrated phase,^22,55^ triggering a phase separation that brings the two phases to the equilibrium. When only one phase is present in the metastability window, the second phase will form by nucleating droplets of the opposite concentration,^56^ as shown in Fig. 4(a).

The same mechanism of uphill diffusion is present in the LLCPS, an isotropic state brought to a metastable state where *ϕ* > 3.34, will nucleate and form domains of nematic and more concentrated solutions. These newly formed domains will grow in volume and concentration until the phase equilibrium is reached.^22^ At the same time, nematic phases at concentrations lower than 4.49 will nucleate isotropic domains, often called negative tactoids.^57^ For unstable states, phase separation is led by spinodal decomposition, which will form new nematic and isotropic states depending on the concentration.^58^ These two behaviors are depicted in Fig. 4(b). Lastly, we notice that the metastable states for the nematic and isotropic fluids are overlapping each other, and both are possible for the same concentrations, between *ϕ*_S_ and *ϕ*_B_. This is a result of the hysteresis behavior concealed in the physics of the Onsager model. Depending on the history of the system, we can have both tactoids nucleating from a supersaturated isotropic solution or negative tactoids in a nematic background, at the same concentration values. We resort to two thought experiments to clarify this behaviour. In the first one, we take a solution of hard rods, whose scaled concentration *ϕ* is just above the isotropic boundary *ϕ*_I_. By fixing the initial order parameter at *s* = 0, we have a supersaturated isotropic solution, where nematic tactoids will nucleate, and eventually grow until phase separation is reached. This has already been observed in many different experimental systems.^25,26,32^ If the initial concentration is higher than *ϕ*_B_ = 4, spinodal decomposition will drive the phase separation, while for *ϕ* > *ϕ*_N_ = 4.49, the fluid will simply change in alignment without undergoing phase separation. See Fig. 4(b). Conversely, if we take a solution in a nematic state, and we decrease the concentration *ϕ* below 4.49, the undersaturated nematic phase will nucleate negative tactoids and start to phase separate. If we bring down the concentration faster than the transport rates typical of nucleation and growth, and reach values lower than 3.659, the nematic state will become unstable and the solution will undergo phase separation through spinodal decomposition. For LLCPS, this other type of metastability is sketched in Fig. 4(b). To summarize: any liquid crystalline system described by the Onsager theory at an initial concentration 3.34 < *ϕ* < 4.49 will phase separate into two phases set at *ϕ*_I_ = 3.34 and *ϕ*_N_ = 4.49, as these are the only two phases equalizing simultaneously chemical potential and osmotic pressure in the two phases. However, the way by which phase separation will proceed, and thus the final morphologies, will depend on the initial state (isotropic or nematic) as well as the initial composition. For an initially isotropic phase such that 3.34 < *ϕ* < 4, the isotropic state is metastable, *i.e.* it exists as a minimum (absolute or relative) on the Onsager excess free energy (see Fig. 2(a)) and phase separation will occur by nucleation and growth of nematic tactoids within an isotropic continuous phase; for 4 < *ϕ* < 4.49, the excess free energy has no longer a minimum at the isotropic phase (*s* = 0), and the isotropic state is unstable leading to spinodal decomposition (see Fig. 4(b)). Reversely, for an initially nematic phase such that 3.659 < *ϕ* < 4.49, the nematic state is metastable, *i.e.* it exists as a minimum (absolute or relative) on the Onsager excess free energy (see Fig. 2(a)) and phase separation will occur by nucleation and growth of isotropic tactoids within a nematic continuous phase; for 3.34 < *ϕ* < 3.659 the excess free energy has no longer a minimum at the nematic state (*s* > 0) phase, the nematic state becomes unstable leading to spinodal decomposition (see Fig. 4(b)).

## 7 Conclusion

To conclude, we reviewed the liquid–liquid phase separation process and the most important thermodynamical properties that are involved in phase separations. Binodal lines and spinodal lines are extremely important boundaries that define the dynamics of phase separation: spinodal lines surround the spinodal region, where the separation is driven by spinodal decomposition, while binodal lines delimit the stable from metastable zones, where nucleation and growth is the mechanism that leads to phase separation. We investigated the difference between liquid–liquid phase separation (LLPS) and the recently introduced liquid–liquid crystalline phase separation (LLCPS). The former is usually described through the theoretical framework of the Flory–Huggins theory, based on the balance between mixing entropy and interaction energy. On the contrary, LLCPS are rationalized through the Onsager theory for hard rods. This theory relies on a purely entropic contribution in the free energy to describe the two different phases, isotropic and nematic. This core difference in the formulation of the thermodynamic energy unveils a completely different underlying physics. In LLPS, when an external parameter *χ* changes, a miscibility gap forms. A certain range of volume fraction *φ* becomes inaccessible. In LLCPS, we can define two states of the liquid: an isotropic and a nematic phase, determined by the orientational order of the molecules. As in LLPS, LLCPS both phases separate *via* nucleation and growth or spinodal decomposition, but while in the first only a change in concentration is observed, in LLCPS the evolution involves both the concentration and the alignment.

## Author contributions

R. M. and P. A. designed the study, the content and wrote the manuscript. P. A. developed the theoretical formalism in the appendix and performed the corresponding analysis. R. M. supervised the project. All authors discussed the results and contributed to the final manuscript.

## Conflicts of interest

There are no conflicts to declare.

## Supplementary Material

## Appendices

### Appendix: A simplified Onsager theory

In this appendix, we briefly report an approximation of Onsager theory. For a more detailed discussion see ref. 22, 24 and 48. The excess free energy density of Onsager theory, that is the part that differentiates it from a perfect gas can be written as a sum of two terms:

12 *f*_O_ = *σ*_1_(*ψ*) + *ϕσ*_2_(*ψ*)

those two quantities depend on the angular distribution, which for Onsager theory is , where *Θ* is the angle and *α* a parameter that controls the intensity of the alignment. For really high values of *α*, when the order parameter tends to 1, we can approximate *ψ* with

13

The above described quantities, *σ*_1_ and *σ*_2_, can be written as

14 *σ*_1_(*α*) ∼ log *α* − 1,

15

From these expressions, we see that *σ*_1_ grows slowly as the log of *α*, while *σ*_2_, the excluded volume term, decreases in magnitude with increasing alignment. If we minimize eqn (12), we obtain:

16

The alignment grows quadratically with the concentration, for nematic phases. From eqn (12), using the above approximations, we can compute the osmotic pressure Π and chemical potential *μ*:

17

and

18

By equating Π_I_ = Π_N_ and *μ*_I_ = *μ*_N_, we obtain

19 *ϕ*_I_ = 3.36 *ϕ*_N_ = 4.68

Those values are sufficiently close to the exact values calculated by Onsager,^48^ where *ϕ*_I_ = 3.34 and *ϕ*_N_ = 4.49.

## References

[cit1] Landau L. D. , Course of Theoretical Physics, Pergamon Press, 1958, vol. 5

[cit2] Stanley H. E. , Phase transitions and critical phenomena, Clarendon Press, Oxford, 1971, vol. 7

[cit3] Quiroz F. G., Fiore V. F., Levorse J., Polak L., Wong E., Pasolli H. A., Fuchs E. (2020). Science.

[cit4] Poole P. H., Grande T., Angell C. A., McMillan P. F. (1997). Science.

[cit5] Gao Z., Fang Y., Cao Y., Liao H., Nishinari K., Phillips G. O. (2017). Food Hydrocolloids.

[cit6] Alberti S., Gladfelter A., Mittag T. (2019). Cell.

[cit7] Dignon G. L., Best R. B., Mittal J. (2020). Annu. Rev. Phys. Chem..

[cit8] Garaizar A., Sanchez-Burgos I., Collepardo-Guevara R., Espinosa J. R. (2020). Molecules.

[cit9] Katayama Y., Mizutani T., Utsumi W., Shimomura O., Yamakata M., Funakoshi K.-I. (2000). Nature.

[cit10] Hyman A. A., Weber C. A., Jülicher F. (2014). Annu. Rev. Cell Dev. Biol..

[cit11] Zhang H., Ji X., Li P., Liu C., Lou J., Wang Z., Wen W., Xiao Y., Zhang M., Zhu X. (2020). Sci. China: Life Sci..

[cit12] Feng Z., Chen X., Wu X., Zhang M. (2019). J. Biol. Chem..

[cit13] Gomes E., Shorter J. (2019). J. Biol. Chem..

[cit14] Boeynaems S., Alberti S., Fawzi N. L., Mittag T., Polymenidou M., Rousseau F., Schymkowitz J., Shorter J., Wolozin B., Van Den Bosch L. (2018). et al.. Trends Cell Biol..

[cit15] Jing H., Bai Q., Lin Y., Chang H., Yin D., Liang D. (2020). Langmuir.

[cit16] Guo Q., Shi X., Wang X. (2021). Non-coding RNA Res..

[cit17] Nott T. J., Craggs T. D., Baldwin A. J. (2016). Nat. Chem..

[cit18] Peng P.-H., Hsu K.-W., Wu K.-J. (2021). Am. J. Cancer Res..

[cit19] Chen H., Cui Y., Han X., Hu W., Sun M., Zhang Y., Wang P.-H., Song G., Chen W., Lou J. (2020). Cell Res..

[cit20] Flory P. J. , Principles of polymer chemistry, Cornell university press, 1953

[cit21] Rubinstein M. and Colby R. H., et al., Polymer physics, Oxford university press, New York, 2003, vol. 23

[cit22] Azzari P., Bagnani M., Mezzenga R. (2021). Soft Matter.

[cit23] Fraccia T. P., Zanchetta G. (2021). Curr. Opin. Colloid Interface Sci..

[cit24] De Gennes P.-G. and Prost J., The physics of liquid crystals, Oxford university press, 1993

[cit25] Bagnani M., Nyström G., De Michele C., Mezzenga R. (2019). ACS Nano.

[cit26] Bagnani M., Azzari P., Assenza S., Mezzenga R. (2019). Sci. Rep..

[cit27] Bagnani M., Azzari P., De Michele C., Arcari M., Mezzenga R. (2021). Soft Matter.

[cit28] Jamali V., Behabtu N., Senyuk B., Lee J. A., Smalyukh I. I., van der Schoot P., Pasquali M. (2015). Phys. Rev. E: Stat., Nonlinear, Soft Matter Phys..

[cit29] Woltman S. J. , Crawford G. P. and Jay G. D., Liquid crystals: frontiers in biomedical applications, World Scientific, 2007

[cit30] Tadwee I., Shahi S., Ramteke V., Syed I. (2012). Int. J. Pharm. Res. Allied Sci..

[cit31] Rey A. D. (2010). Soft Matter.

[cit32] Nyström G., Arcari M., Mezzenga R. (2018). Nat. Nanotechnol..

[cit33] Collins S. R., Douglass A., Vale R. D., Weissman J. S., Eisenberg D. (2004). PLoS Biol..

[cit34] Cao Y., Mezzenga R. (2019). Adv. Colloid Interface Sci..

[cit35] Wei G., Su Z., Reynolds N. P., Arosio P., Hamley I. W., Gazit E., Mezzenga R. (2017). Chem. Soc. Rev..

[cit36] Chiti F., Dobson C. M. (2006). et al.. Annu. Rev. Biochem..

[cit37] Gallo P. and Rovere M., Physics of Liquid Matter, Springer, 2021

[cit38] de Gennes P.-G. (1980). J. Chem. Phys..

[cit39] Ma T. and Wang S., Phase transition dynamics, Springer, 2014

[cit40] Flory P. J. (1942). J. Chem. Phys..

[cit41] Yamamoto T., Narita T., Nobe M., Dobashi T. (2004). Macromolecules.

[cit42] Kamide K. , Polymer Science Library, 1990, vol. 9

[cit43] Qian D., Michaels T. C., Knowles T. P. (2022). J. Phys. Chem. Lett..

[cit44] Cheng S. Z., Keller A. (1998). Annu. Rev. Mater. Res..

[cit45] Guckenheimer J. and Holmes P., Nonlinear oscillations, dynamical systems, and bifurcations of vector fields, Springer Science & Business Media, 2013, vol. 42

[cit46] Stauffer D., Ferer M., Wortis M. (1972). Phys. Rev. Lett..

[cit47] Hamley I. W. (2010). Soft Matter.

[cit48] Onsager L. (1949). Ann. N. Y. Acad. Sci..

[cit49] Kayser Jr R. F., Raveché H. J. (1978). Phys. Rev. A: At., Mol., Opt. Phys..

[cit50] Vollmer M. A. (2017). Arch. Ration. Mech. Anal..

[cit51] Koechner W. , Solid-state laser engineering, Springer, 2013, vol. 1

[cit52] Aharoni A. , et al., Introduction to the Theory of Ferromagnetism, Clarendon Press, 2000, vol. 109

[cit53] Borštnik A., Žumer S. (1997). Phys. Rev. E: Stat., Nonlinear, Soft Matter Phys..

[cit54] Hohenberg P., Swift J. (1995). Phys. Rev. E: Stat., Nonlinear, Soft Matter Phys..

[cit55] Krishna R. (2015). Chem. Soc. Rev..

[cit56] Yuan C., Levin A., Chen W., Xing R., Zou Q., Herling T. W., Challa P. K., Knowles T. P., Yan X. (2019). Angew. Chem., Int. Ed..

[cit57] Nastishin Y. A., Liu H., Schneider T., Nazarenko V., Vasyuta R., Shiyanovskii S., Lavrentovich O. (2005). Phys. Rev. E: Stat., Nonlinear, Soft Matter Phys..

[cit58] Reyes C. G., Baller J., Araki T., Lagerwall J. P. (2019). Soft Matter.

